# Reversed-Phase Medium-Pressure Liquid Chromatography Purification of Omega-3 Fatty Acid Ethyl Esters Using AQ-C18

**DOI:** 10.3390/md22060285

**Published:** 2024-06-19

**Authors:** Mingxin Sang, Nan Pan, Jingna Wu, Xiaoting Chen, Shuilin Cai, Huan Fang, Meitian Xiao, Xiaoming Jiang, Zhiyu Liu

**Affiliations:** 1 College of Chemical Engineering, Huaqiao University, Xiamen 361021, China; 22014087026@stu.hqu.edu.cn (M.S.); mtxiao@hqu.edu.cn (M.X.); 2 Fisheries Research Institute of Fujian, Xiamen 361013, China; xtchen@jmu.edu.cn (X.C.); caishuilin@hqu.edu.cn (S.C.); 220820081@fzu.edu.cn (H.F.); 3 Xiamen Key Laboratory of Marine Medicinal Natural Products Resources, Fujian Universities and Colleges Engineering Research Center of Marine Biopharmaceutical Resources, Xiamen Medical College, Xiamen 361023, China; wjn@xmmc.edu.cn; 4 College of Biological Science and Engineering, Fuzhou University, Fuzhou 350108, China; 5 Quanzhou Institute of Marine Bioresources Industry, Quanzhou 362000, China; jxm@ouc.edu.cn; 6 Key Laboratory of Cultivation and High-Value Utilization of Marine Organisms in Fujian Province, National Research and Development Center for Marine Fish Processing (Xiamen), Xiamen 361013, China

**Keywords:** omega-3 fatty acids, eicosapentaenoic acid, docosahexaenoic acid, reverse-phase medium-pressure liquid chromatography, AQ-C18, high-purity

## Abstract

Omega-3 fatty acids are in high demand due to their efficacy in treating hypertriglyceridemia and preventing cardiovascular diseases. However, the growth of the industry is hampered by low purity and insufficient productivity. This study aims to develop an efficient RP-MPLC purification method for omega-3 fatty acid ethyl esters with high purity and capacity. The results indicate that the AQ-C18 featuring polar end-capped silanol groups outperformed C18 and others in retention time and impurity separation. By injecting pure fish oil esters with a volume equivalent to a 1.25% bed volume on an AQ-C18 MPLC column using a binary isocratic methanol–water (90:10, *v*:*v*) mobile phase at 30 mL/min, optimal omega-3 fatty acid ethyl esters were obtained, with the notable purity of 90.34% and a recovery rate of 74.30%. The total content of EPA and DHA produced increased from 67.91% to 85.27%, meeting the acceptance criteria of no less than 84% set by the 2020 edition of the Pharmacopoeia of the People’s Republic of China. In contrast, RP-MPLC significantly enhanced the production efficiency per unit output compared to RP-HPLC. This study demonstrates a pioneering approach to producing omega-3 fatty acid ethyl esters with high purity and of greater quantity using AQ-C18 RP-MPLC, showing this method’s significant potential for use in industrial-scale manufacturing.

## 1. Introduction

Omega-3 fatty acids are long-chain poly-unsaturated fatty acids that feature an initial conjugated double bond at the third carbon atom from the methyl end; principally the eicosapentaenoic acid (C20:5 ω-3; EPA) and docosahexaenoic acid (C22:6 ω-3; DHA) are of major interest [1], which are derived from α-linolenic acid (C18:3 ω-3) via a series of chain elongations and desaturations. However, due to insufficient Δ-12 and Δ-15 desaturases for conversion in the human body [2,3], omega-3 fatty acids have to be obtained via dietary means [4] and are considered essential to human health [5,6]. An increased consumption of EPA and DHA has been scientifically proven to be beneficial in the treatment and prevention of atherosclerosis [7], myocardial infarction [8], inflammation [9], arthritis [10], diabetes [11], infant brain development [12,13], and cancers [14]. Notably, many epidemiological, observational, and clinical studies have emphasized the efficacy of omega-3 fatty acids in reducing plasma triglyceride levels and preventing cardiovascular diseases [3]. As a result, the authorities that oversee the pharmaceutical industry have set standards for “Omega-3-acid ethyl ester”, “Ethyl polyenoate”, and correlative capsules and thus approved several prescription drugs for treating hypertriglyceridemia or preventing cardiovascular diseases, such as Lovaza^®^ and Omacor^®^ [46% EPA ethyl esters (EPA-EE) + 38% DHA ethyl esters (DHA-EE)], and Vascepa^®^ (96% EPA-EE) [15]. It is recommended by heart associations worldwide that a prescription of omega-3 fatty acids (EPA+DHA or EPA only) at a dose of 4 g/day (>3 g/day total EPA + DHA) represents an effective therapeutic agent in reducing triglycerides. With this increasing attention, the demand for high-purity omega-3 fatty acid has surged significantly. However, prolonged overfishing has led to a sharp decline in the main fish sources, resulting in a rapid growth in the price of omega-3 fatty acid. Nevertheless, there are only a handful of companies worldwide with the capacity to manufacture pharmaceutical-grade omega-3 fatty acid [16]. Hence, it is necessary to develop a universally applicable and cost-effective technology that ensures the safe production of high-purity omega-3 fatty acid.

Omega-3 fatty acid ethyl esters have been concentrated using various techniques, including low-temperature crystallization [17,18], urea inclusion [19,20], molecular distillation [21,22], lipase catalysis [23,24], and supercritical fluid extraction [25]. However, the purity of the omega-3 fatty acids produced using these methods does not meet the standards of the Pharmacopoeia. Chromatography is considered the primary purification technology used in the industrial-scale manufacturing of commercially valuable compounds with high purity, including high-pressure liquid chromatography (HPLC) [26], high-speed counter-current chromatography (HSCCC) [27], supercritical fluid chromatography (SFC) [28], and simulated moving bed (SMB) chromatography [29]. These methods, which rely on high-pressure conditions, effectively improve the purity of omega-3 fatty acids [30,31]. However, several drawbacks to these methods hinder their broader application in the industry, such as the requirement for relatively high initial sample purity, high energy consumption, complex equipment requirements, and high costs during production. The HPLC method utilizes packings with a smaller particle size and has a lower sample-loading volume to ensure high resolution and sensitivity [30]. The SFC method leverages supercritical fluids of CO_2_ as the mobile phase, necessitating precise control over the operating parameters (pressure and temperature), which often requires specialized expertise [31]. SMB chromatography is currently acknowledged for its continuous operation and enhanced efficiency in the purification process [32]; however, for SMB to be effective, a high concentration of raw materials is required, and it utilizes smaller-particle stationary phases to ensure effective purification, which consequently elevates the cost. Therefore, developing a cost-effective, high-throughput chromatographic method tailored to low-purity fish oil samples to produce omega-3 fatty acids with high purity is of significant importance.

Reversed-phase medium-pressure liquid chromatography (RP-MPLC) is a type of liquid chromatography that operates at pressures ranging between 0 and 200 psi; the main features of RP-MPLC include a fast separation speed and high efficiency [33]. With significantly reduced instrumentation and operational expenses relative to HPLC, SFC, and SMB, the cost-effective alternative of RP-MPLC operates under medium pressure and employs packing materials with larger particle sizes, which leads to larger sample preparation capacities, reduces the impact of impurities on the packing materials, and shortens the processing times. By accepting lower-purity materials and decreasing the frequency of preliminary purification stages, RP-MPLC plays an important role in the separation and purification of saponins [34], polysaccharides [35], and polypeptides [36] and has gradually become a popular method in recent years [35,37]. Additionally, Ishihara et al. [38] purified stearidonic acid (C18:4 ω-3) and hexadecatetraenoic acid (C16:4 ω-3) to more than 95% purity using RP-MPLC. These results indicate that RP-MPLC could potentially produce high-purity omega-3 fatty acid ethyl esters.

In this study, RP-MPLC was initially employed to deliver high-purity omega-3 fatty acid ethyl esters, targeting a total content of EPA and DHA of not less than (NLT) 84%, as stipulated by the Pharmacopoeia. Fundamental variables controlling separation were evaluated and optimized based on the purity and recovery rate, including the packing material, mobile phase, sample volume, sample concentration, flow rate, and mobile-phase composition. Furthermore, comparisons were made between RP-MPLC and RP-HPLC using the same packings, with the aim of assessing the effectiveness of the purification. Ultimately, this research provides a new approach and theoretical basis for the cost-effective and high-throughput production of high-purity omega-3 fatty acids on an industrial manufacturing scale.

## 2. Results and Discussion

### 2.1. Fatty Acid Composition of Fish Oil Ethyl Esters

**Figure 1:**
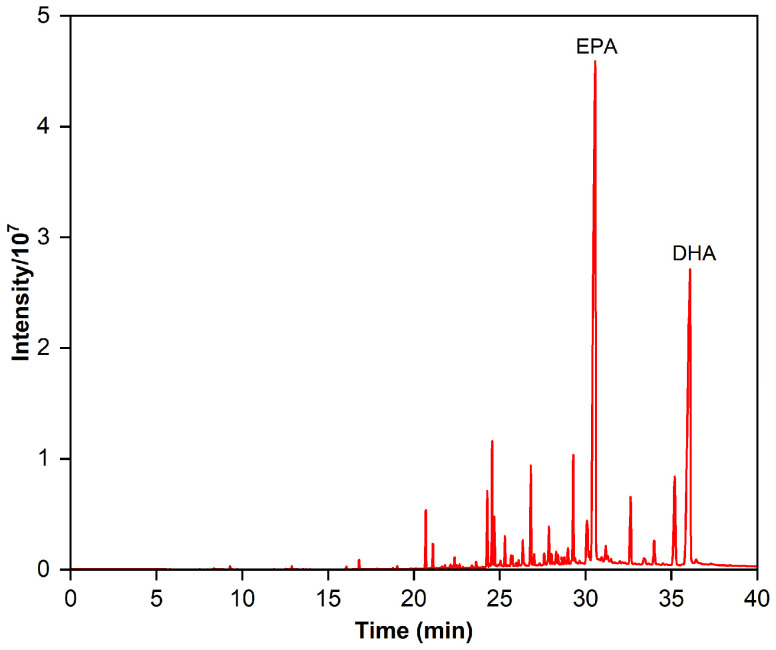
Gas chromatogram of the fish oil ethyl esters.

Identifying fatty acids and esters with a similar equivalent carbon length from the EPA and DHA is a crucial stage in the development of the RP-MPLC method. The GC chromatogram for the fish oil ethyl esters is displayed in Figure 1, and the fatty acid composition is outlined in Table 1. We can see that 24 fatty acids were detected in the esters, including 6 saturated fatty acids (SFAs), 5 mono-unsaturated fatty acids (MUFAs), and 13 poly-unsaturated fatty acids (PUFAs), accounting for 4.45%, 7.84%, and 87.71% of the content, respectively. The major SFAs were C18:0 and C16:0, at 2.18% and 1.52%, respectively. The primary MUFAs were C18:1n9, C22:1n9, and C20:1n9, at 4.64%, 1.19%, and 1.17%, respectively. PUFAs made up the bulk of fatty acids, with ω-6 PUFAs at 9.12% (mainly C20:4n6, C18:4n6, and C22:5n6) and ω-3 PUFAs at a higher percentage, predominantly EPA (C20:5n3), DHA (C22:6n3), DPA (C22:5n3), C21:5n3, and C20:3n3. However, the sum of the content of EPA and DHA was 67.91%, falling short of the acceptance criteria of the Chinese Pharmacopoeia (2020 edition) for “Ethyl polyenoate”, which require NLT 84% purity of the total content of EPA and DHA.

### 2.2. Fatty Acid Composition of Separate Fractions in RP-MPLC Chromatogram

**Figure 2:**
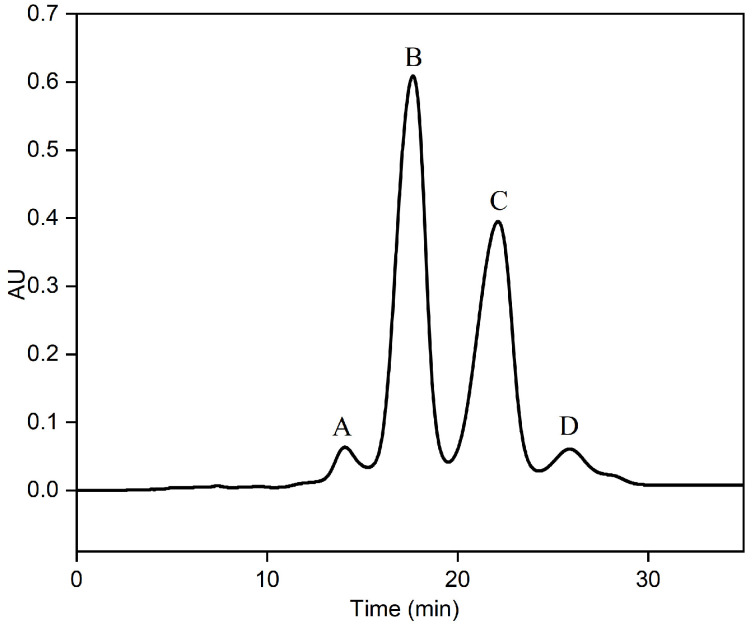
RP-MPLC chromatogram of fish oil ethyl esters with four fractions: A, B, C, and D. Conditions: stationary phase, C18; sample load, 0.4 mL fish oil ethyl esters; mobile phase, methanol–water (90:10, *v:v*); flow rate, 20 mL/min; detector, UV 210 nm; room temperature.

**Figure 3:**
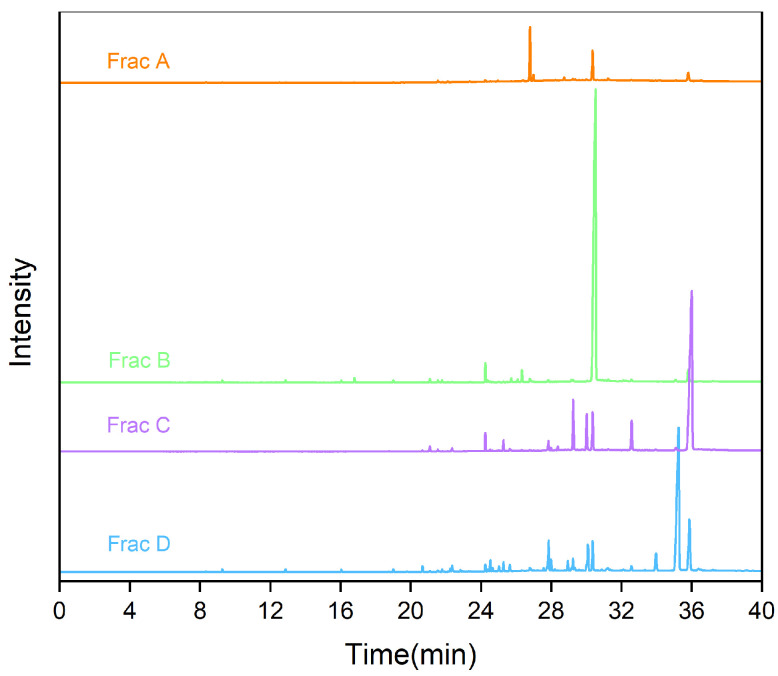
Gas chromatogram of fractions A, B, C, and D.

**Figure 4:**
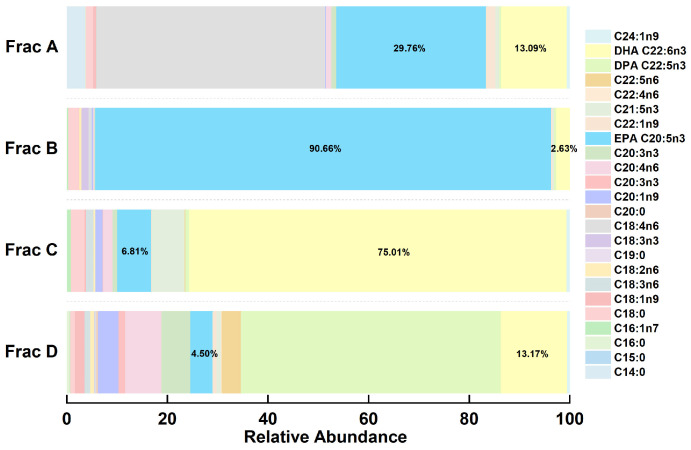
Stacked histograms of fatty acid compositions from different fractions.

An RP-MPLC chromatogram of fish oil ethyl esters is shown in Figure 2. Four major fractions (A–D), corresponding to the separated group, were collected, vacuum-evaporated, and analyzed using GC-MS. A GC chromatogram of the fish oil ethyl esters, with four fractions (A–D), is displayed in Figure 3. The varying fatty acid compositions across different fractions are demonstrated in Figure 4.

Fraction A comprised a total of 42.85% of EPA (29.76%) and DHA (13.09%), with the remainder predominantly made up of C18:4n6 (45.34%), C14:0 (3.76%), C18:0 (1.44%), and C20:4n6 (1.01%). Conversely, fraction B was mainly represented by EPA, at 90.66%, with the remainder mostly including DHA (2.63%), C18:0 (2.11%), and C18:3n3 (1.42%). Fraction C displayed the highest DHA content, at 75.01%, complemented by EPA (6.81%), C21:5n3 (6.48%), C18:0 (2.70%), C20:4n6 (1.90%), C18:3n6 (1.49%), and C20:1n9 (1.39%). Fraction D was notable for the highest DPA concentration, at 51.70%, with the rest primarily comprising DHA (13.17%), C20:4n6 (7.27%), C20:3n3 (5.67%), C20:5n3 (4.50%), C20:1n9 (4.10%), C22:5n6 (3.84%), and C18:1n9 (1.88%). In essence, fraction B and fraction C were identified as the target components of EPA and DHA, respectively, which enabled their precise collection for the further purification of omega-3 fatty acid ethyl esters.

### 2.3. Optimization of RP-MPLC

#### 2.3.1. Effects of Packing Materials

**Figure 5:**
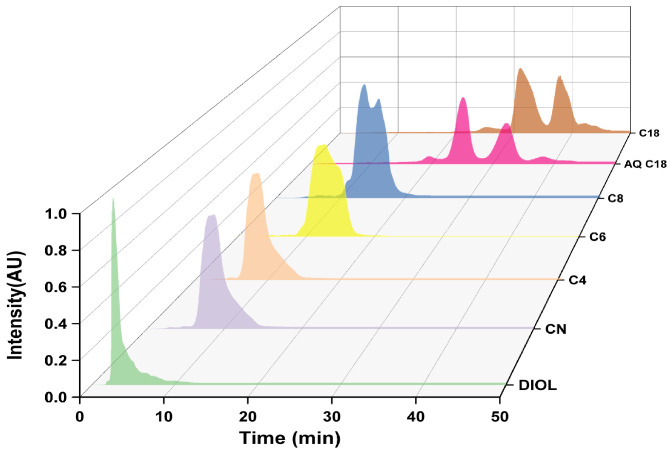
RP-MPLC chromatograms of omega-3 fatty acid ethyl esters using various packing materials.

The packing materials are the “heart” of the chromatographic system. The physicochemical properties of the packing materials, including the uniformity of the packing structure (monolithic, porous, or nonporous), the geometry (particle size, area of the bed, and pore size and shape), and the type of attached ligands, significantly influence the separation efficacy [5,39]. To identify column packings that provide high throughput, low back pressure, strong sensitivity, and high resolution for efficient separation, various bonded packing materials (CN, Diol, C4, C6, C8, C18, and AQ-C18) were evaluated in the purification of omega-3 fatty acid ethyl esters (Figure 5 and Table 2).

Analysis of the elution curves for CN, Diol, C4, C6, and C8 revealed that EPA and DHA did not acquire baseline separation from the preceding and subsequent impurities. CN, a normal-phase stationary phase with cyanide groups, was more suited to the reverse-phase separation of weakly polar compounds, showing a weak affinity for EPA and DHA [40]. Diol, with intermediate polarity, and C4, C6, and C8, with shorter carbon chains, exhibited weaker affinity, leading to shorter retention times and less effective purification of EPA and DHA. Thus, these materials were deemed unsuitable.

**Figure 6:**
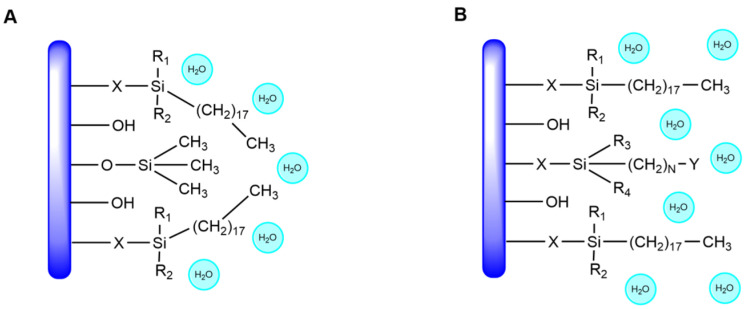
Structural differences between RP-MPLC stationary phases (**A**) C18 and (**B**) AQ-C18.

In contrast, the elution curves of C18 and AQ-C18 packing materials display clear target peaks for EPA and DHA, with good separation from adjacent impurity peaks. AQ-C18 demonstrates earlier peak emergence, shorter purification time, and lower solvent consumption compared to C18, showcasing superior separation efficiency. Both C18 and AQ-C18 are non-polar reversed-phase stationary phases with octadecyl carbon chains bonded to silica. However, AQ-C18 undergoes the polar end-capping of silica hydroxyl groups, minimizing the residual silanol on the surface [41] and thus enhancing the separation effects of omega-3 fatty acid ethyl esters (Figure 6). Based on these findings, AQ-C18 was chosen as the stationary phase for subsequent experiments.

#### 2.3.2. Effects of Mobile Phases

**Figure 7:**
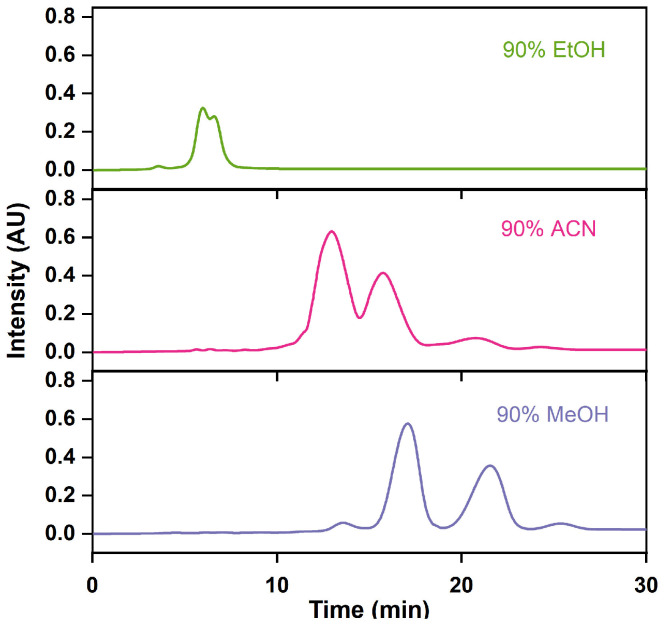
RP-MPLC chromatograms of omega-3 fatty acid ethyl esters via different mobile phases.

The appropriate mobile phase plays an important auxiliary role in improving separation efficiency. When choosing the mobile phase for industrial applications, emphasis has been placed on the separation efficacy, cost, column pressure drop, and ease of subsequent product purification [42]. As a result, solvents characterized by low viscosity, low boiling points, and low cost are preferred. In Figure 7 and Table 3, we can see that ethanol and acetonitrile were ineffective in achieving the baseline separation of impurities from omega-3 fatty acids, unlike methanol, which was successful. Despite its higher viscosity, methanol’s lower boiling point facilitated easier separation from the product than acetonitrile and ethanol. More importantly, methanol is an effective solvent, with easy recovery from boiling point [43,44], which allows resource reuse and promotes the development of the circular economy [45]. Consequently, methanol was employed as the preferred mobile phase.

#### 2.3.3. Effects of Sample Load Volume

**Figure 8:**
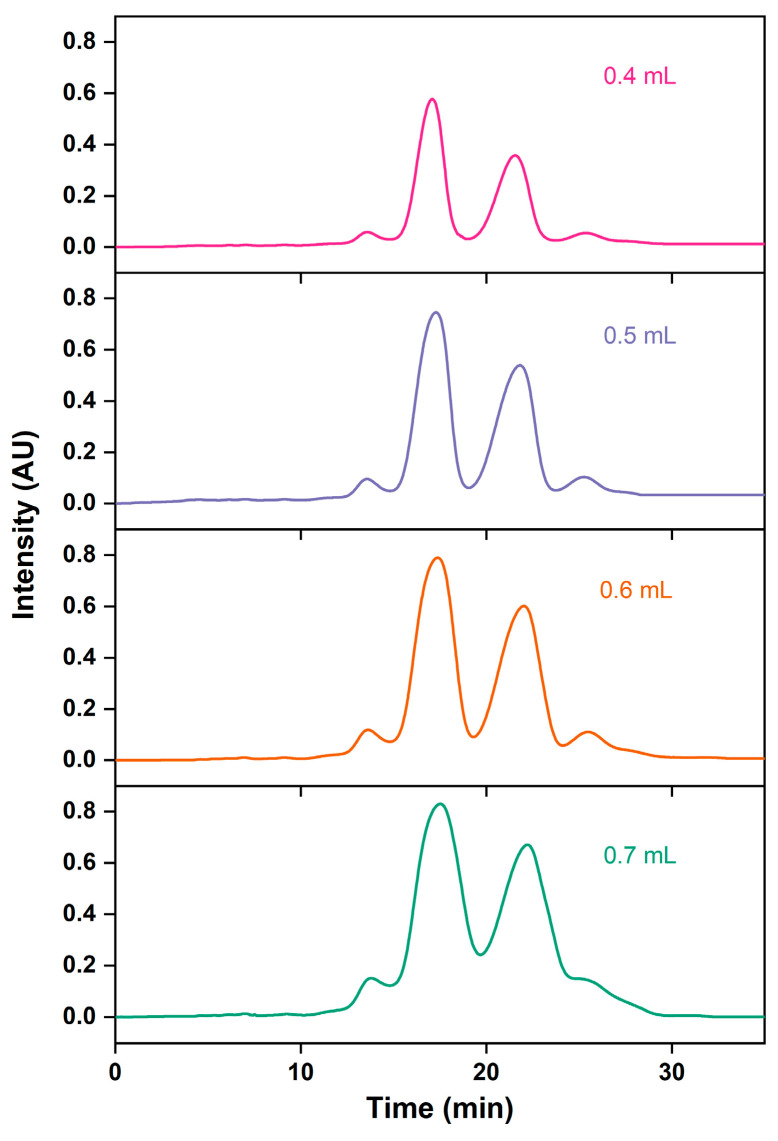
RP-MPLC chromatograms of omega-3 fatty acid ethyl esters with different injection volumes.

According to the nonlinear theory of chromatographic preparation, increasing the sample volume can improve the processing capacity of chromatography, boost the product recovery rate, and enhance production efficiency [46,47]. As shown with the growth loading volume in Table 4 and Figure 8, the retention time is delayed, peak shapes are widened, the resolution is reduced, and the purification time is increased. This is consistent with the experimental findings of Dillon et al. [48]. This could be due to the adsorption of more impurities on the AQ-C18 packings, which affects the separation of main and impurity peaks, thereby reducing the purity of the target substance [49]. With a sample volume of 0.6 mL, the recovery rates for the total ethyl esters for the EPA and DHA peak were the highest (83.57%). To maximize the loading volume while achieving better separation effects, the sample loading of 0.6 mL, equivalent to the 1.25% bed volume of the chromatographic column, was selected.

#### 2.3.4. Effects of Sample Concentration

**Figure 9:**
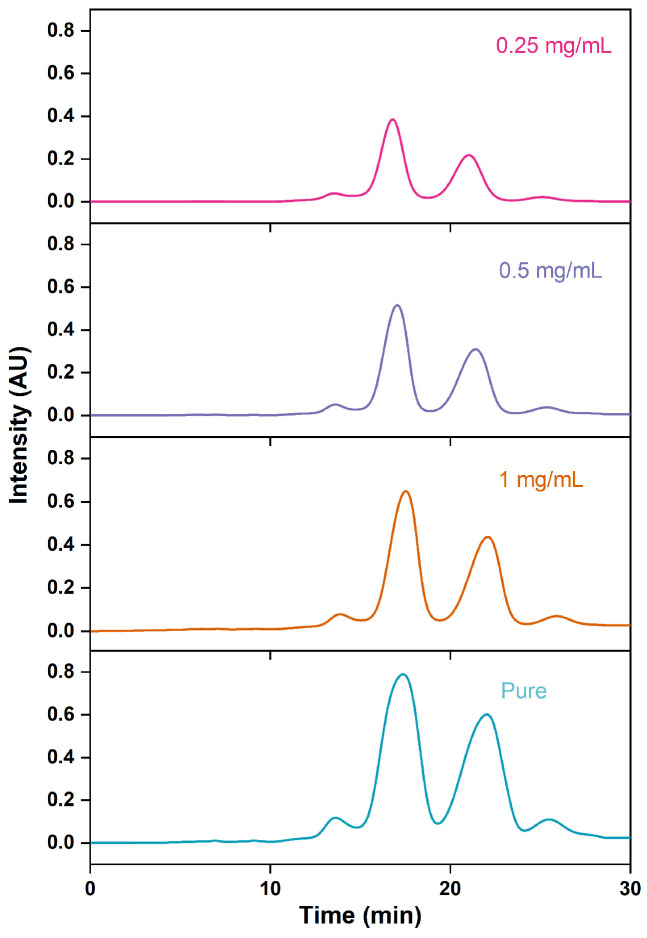
RP-MPLC chromatograms of omega-3 fatty acid ethyl esters of different concentrations.

During industrial production, increasing the sample concentration can enhance the chromatographic processing capacity, while reducing the concentration helps to promote the partition and adsorption processes of analytes onto chromatographic packing material, thus enhancing the separation between target substances and impurities [50,51,52,53]. However, this improvement comes at the cost of a corresponding decrease in recovery rates. Chromatographic curves depicting various concentrations of fish oil ethyl esters diluted with methanol in RP-MPLC are presented in Figure 9, accompanied by Table 5. As the concentrations of fish oil ethyl esters increased, the purity of ethyl esters of EPA and DHA decreased, whereas the recovery rate, retention time, and resolution exhibited an increase. Conversely, the use of pure fish oil for injection reduced the resolution of ethyl esters of EPA and DHA, achieving a separation factor of 1.23 for front impurities and 1.10 for rear impurities, with a purity of 85.75%. The recovery rate of ethyl esters of EPA and DHA steadily increased with the sample concentration, peaking at 74.62% with pure fish oil. To maximize production efficiency, the pure fish oil ethyl esters were chosen.

#### 2.3.5. Effects of Flow Rate

**Figure 10:**
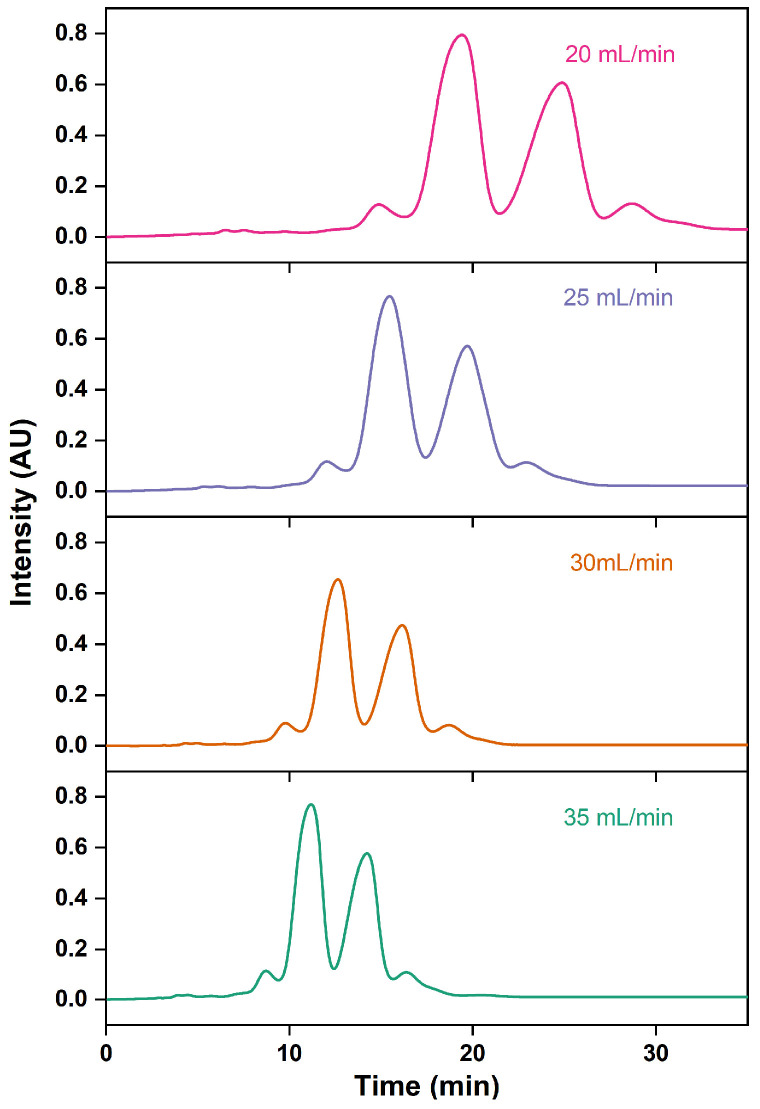
RP-MPLC chromatograms of omega-3 fatty acid ethyl esters with different flow rates.

In a liquid chromatography system, increasing the flow rate can shorten the elution time of analytes, albeit with the potential downside of compromising the robustness of chromatographic analysis [54,55]. As the flow rate accelerates, the peak elution time shifts earlier and the peak shape becomes more compact, resulting in closer proximity between the peaks of the ethyl esters EPA and DHA and the surrounding impurity peaks (Figure 10). As the flow rate speeds up, the elution time of the ethyl esters EPA and DHA consistently decreases, leading to a reduced retention time and resolution and thus causing a decline in purity (Table 6). Additionally, the recovery rates of the ethyl esters EPA and DHA reduce continuously with the growth in flow rate. This is similar to the trends observed by Oh et al. [30], which could be attributed to the fact that excessively high flow rates might impede the adsorption of target compounds to the stationary phase. To optimize the separation efficiency while minimizing time and solvent usage, the flow rate of 30 mL/min was selected.

#### 2.3.6. Effects of Mobile-Phase Composition

**Figure 11:**
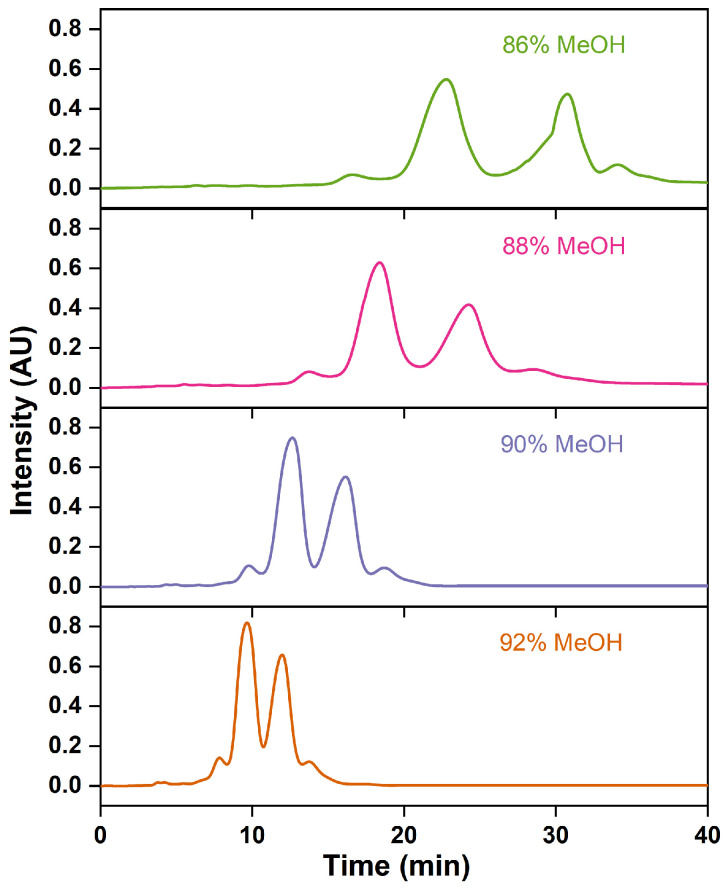
RP-MPLC chromatograms of omega-3 fatty acid ethyl esters with different mobile-phase compositions.

The proportion of the organic solvent in the mobile phase modifies its polarity, thereby altering the distribution coefficient of the sample components in the stationary phase and affecting the separation efficiency [56]. Increasing the methanol proportions advances the peak emergence time, broadens the peak shape, and reduces the retention time, resolution, and purity of the ethyl esters EPA and DHA (Figure 11 and Table 7). This is attributed to the fact that increasing the polarity of the mobile phase has been found to improve the separation efficiency by delaying the retention time of the non-polar FAEE in the column [30,48]. With a methanol proportion of 86% to 90%, the purity of omega-3 fatty acid gradually declines; meanwhile, the recovery rate improves. Wei et al. [29] observed the same experimental trend. At a 92% methanol proportion, the purity of ethyl esters of EPA and DHA falls to 83.39%, which does not meet the national Pharmacopoeia standards. Methanol proportions over 90% are not conducive to the preparation of high-purity omega-3 fatty acids. Therefore, a 90% methanol solution was chosen as the composition of the mobile phase.

### 2.4. Fatty Acid Analysis of Purified Omega-3 Fatty Acid Ethyl Esters

**Figure 12:**
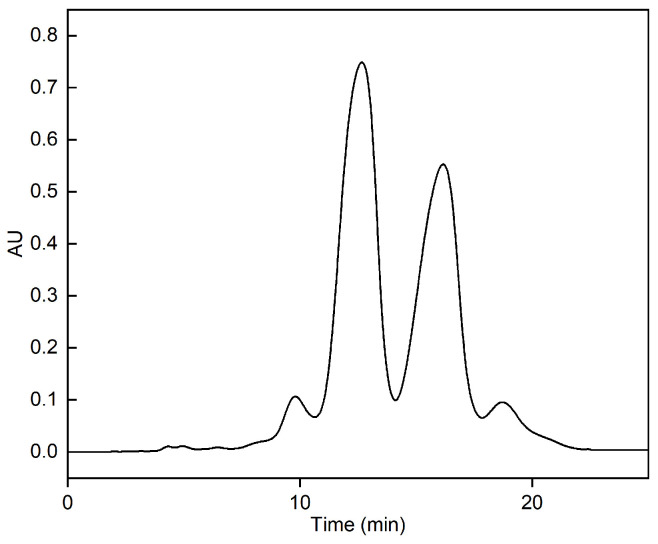
RP-MPLC chromatogram of fish oil ethyl ester under optimal purification conditions. Conditions: stationary phase, AQ-C18; sample load, pure fish oil ethyl esters with volume equivalent to 1.25% bed volume; mobile phase, methanol–water (90:10, *v*:*v*); flow rate, 30 mL/min; detector, UV 210 nm; room temperature.

**Figure 13:**
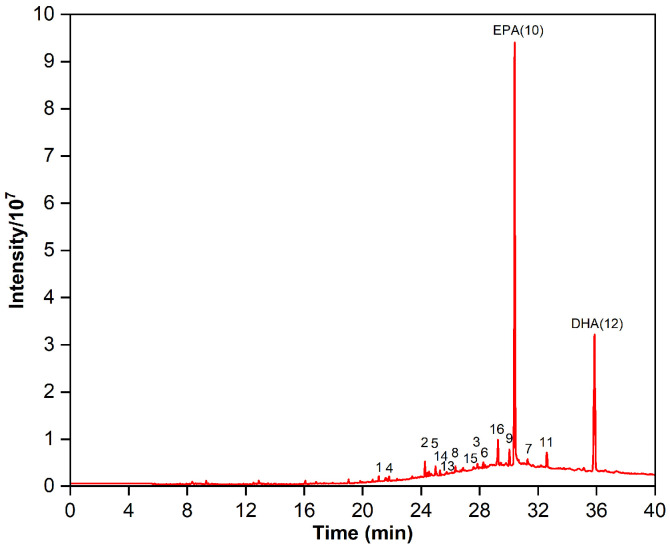
Gas chromatogram of the purified omega-3 fatty acid ethyl esters.

Fish oil ethyl esters equivalent to 1.25% of the column volume were introduced into an RP-MPLC column equipped with AQ-C18 packings (20–40 μm particle size, 100 Å pore size, 320–340 m^2^/g surface area) and eluted with a methanol–water (90:10, *v*:*v*) isocratic mobile phase at a flow rate of 30 mL/min under an operating pressure of 1–4 bar. The RP-MPLC chromatogram of fish oil ethyl esters under optimal purification conditions is shown in Figure 12. Fractions B and C were collected, concentrated under reduced pressure, and subsequently characterized via GC-MS. In total, 16 types of fatty acids were identified in the form of 2.39% SFAs, 3.14% MUFAs, and 94.47% PUFAs (Figure 13 and Table 8). Compared with Table 1, SFAs C14:0, C15:0, and C19:0; MUFA C24:1n9; and PUFAs C22:5n3, C20:3n6, and C22:5n6 were removed. The proportion of ω-3 PUFAs rose from 78.59% to 90.34%, the main components of which were EPA and DHA, accounting for 57.13% and 28.14%, respectively, and totaling 85.27%. Thus, the omega-3 fatty acid ethyl esters purified via AQ-C18 RP-MPLC meet the criteria for “Ethyl polyenoate” in the 2020 edition of the Pharmacopoeia, which states that the combined EPA and DHA content must be at least 84%.

### 2.5. Comparison of RP-MPLC and RP-HPLC

The purification of omega-3 fatty acid ethyl esters using RP-MPLC and RP-HPLC methods was evaluated using the same packing of AQ-C18, focusing on the purity, recovery rate, retention time, operation duration, and solvent consumption. The results are compared in Table 9; both the RP-MPLC and RP-HPLC methods achieved a purity ≥ 84% of the total content of the EPA and DHA ethyl esters, meeting the standards set by the Pharmacopoeia. This indicates that both methods are suitable for purification. Despite RP-MPLC exhibiting a slightly lower purity (85.27%) and recovery rate (74.30%) compared to RP-HPLC, it demonstrated notable advantages in shortening times for the ethyl esters EPA and DHA and the overall operation duration, with reductions of 58.35%, 63.47%, and 70.67%, respectively. Within the permissible operating pressure, the flow rate of RP-MPLC was ten-fold faster than that of RP-HPLC, which significantly improved the separation efficiency, leading to earlier peak emergence and reducing the purification time to one-third of that of RP-HPLC. However, this came at the cost of consuming 1.93 times more solvent. In industrial settings, solvents like methanol and ethanol can be recycled via dehydration with 3Å molecular sieves and rotary evaporation. This helps to cut costs and reduce environmental pollution [57]. In conclusion, when using AQ-C18 as the stationary phase, RP-MPLC not only meets the Pharmacopoeia purity standards for the ethyl esters EPA and DHA, but also enhances the unit time production efficiency by requiring only 29.33% of the purification time compared to RP-HPLC. Additionally, solvent recycling reduces costs, making RP-MPLC more cost-effective in terms of equipment, operation, and maintenance than RP-HPLC. Therefore, RP-MPLC emerges as the preferred method for the large-scale production of omega-3 fatty acids with high purity and yield.

### 2.6. Summary and Prospect of RP-MPLC

Liquid chromatography is highly effective in separating structurally similar fatty acids and can be used to prepare high-purity omega-3 fatty acids [58,59,60,61], as demonstrated in this study. The RP-MPLC method ensures a safer and more reliable omega-3 fatty acid product. This process utilizes AQ-C18 as the stationary phase and methanol as the mobile phase, effectively preventing hazardous reagents such as silver mercury ions and acetone from contaminating the product and thereby eliminating potential risks to human health [62]. The RP-MPLC method requires packings with larger particle sizes and operation at lower pressures, thus allowing for increased sample loading. This enhances the chromatographic processing capacity and results in higher production efficiency. RP-MPLC is an ideal technology for enhancing the purity of substances with commercial value, especially those with low initial purity levels, such as the fish oil ethyl esters studied here, which initially had a combined EPA and DHA purity of only 67.91%. This method uses simpler purification equipment, and its cost-effectiveness is a notable advantage, making it an attractive option for the large-scale industrial purification of omega-3 fatty acid ethyl esters. Furthermore, the applications of RP-MPLC in conjunction with other chromatographic packing materials in separation and purification have been extensively studied [63,64]. It is essential to explore the purification mechanism for improving the efficiency of RP-MPLC.

## 3. Materials and Methods

### 3.1. Materials and Reagents

Fish oil ethyl esters, with an EPA purity of 40.83% and a DHA purity of 27.08%, were kindly provided by Fujian Coland Marine Bioengineering Co., Ltd (Fuzhou, China). Guaranteed-grade sodium chloride and anhydrous sodium sulfate were purchased from Xiya Chemical Technology Co., Ltd (Linyi, China). HPLC-grade methanol, ethanol, acetonitrile, and n-hexane; GC-grade boron trichloride–methanol solution (15% in methanol); standards of EPA ethyl and DHA ethyl; and the standard mixture of 40 fatty acid methyl esters were purchased from ANPLE Laboratory Technologies Inc. (Shanghai, China). Milli-Q water (Integral-3, Merck Millipore, Darmstadt, Germany) was used. All other chemicals were of chromatographic and analytical grade.

### 3.2. RP-MPLC Procedure

**Figure 14:**
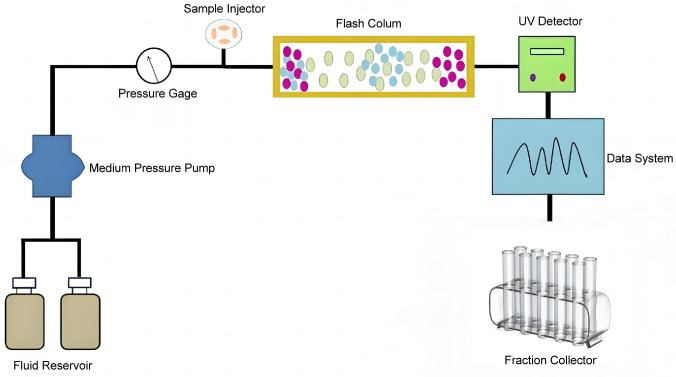
Schematic diagram of experimental apparatus.

A Pure Chromatography Instrument C-815 Flash (Buchi, Switzerland) equipped with two binary gradient pumps and flashpure columns (26.2 mm × 152.3 mm) filled with various stationary phases (20–40 μm particle size, 100 Å pore size, 320–340 m^2^/g surface area) was utilized for the purification of omega-3 fatty acid (Figure 14). Fish oil ethyl esters (equivalent to 0.8–1.4% of column volume) were injected manually through a sample loading loop and mixed with the mobile phase at an electric port valve. The eluent signal was recorded by UV detectors, and peak fractions with signals over 0.05 AU were collected, dried under reduced pressure for solvent removal using an R-100 rotary evaporator (Buchi, Switzerland), and subsequently characterized using a GCMS-QP2020NX gas chromatograph–mass spectrometer (Shimadzu, Japan).

### 3.3. Fatty Acid Analysis

The fatty acid composition of fish oil ethyl esters or RP-MPLC fraction aliquots was analyzed using GC-MS fitted with an SH-Wax capillary column (30 m × 0.25 mm × 0.25 μm). Helium (99.999%) was employed as the carrier gas at a constant flow rate of 3.0 mL/min; the injector temperature was set at 240 °C; the injection mode was splitless; and the pressure was maintained at 109.1 kPa, with a total flow rate of 30 mL/min and a column flow rate of 1.46 mL/min. The injector temperature was set at 230 °C. The temperature program was initiated at 100 °C and held for 3 min, followed by an increase at a rate of 5 °C/min to 230 °C, and then maintained for 15 min. MS detection was set to electron impact (EI) ionization mode, the ion source temperature was set at 230 °C, the interface temperature was 250 °C, the ionization energy was 70 eV, the detector voltage was 0.9 kV, the solvent delay time was 5 min, the mass scan range was set from 35 to 500 *m*/*z*, and the acquisition mode was set to scan.

The ethyl esters were identified by their retention times compared with a fatty acid methyl ester standard mixture. The relative purity (%) of various esters was calculated using integrated peak areas according to Equation (1):

(1) $$P_{i}=\frac{A_{SI}\times F_{FAEEi\text{-}FAi}}{\sum A_{SI}\times F_{FAEEi\text{-}FAi}}\;\times 100\%$$

where *P_i_* represents the percentage of a specific fatty acid in the sample relative to the total fatty acids as a percentage (%), *A_SI_* is the peak area of each fatty acid ethyl ester in the sample, *F_FAEEi_-F_A_*_i_ refers to the coefficient for converting fatty acid ethyl esters to fatty acids, and *∑A_SI_* denotes the sum of peak areas of all fatty acid ethyl esters in the sample.

### 3.4. Optimization of RP-MPLC

The column packing materials (CN, Diol, C4, C6, C8, C18, AQ-C18), sample load volume (0.4 mL, 0.5 mL, 0.6 mL, and 0.7 mL), sample concentration (pure fish oil, 1 g/mL, 0.5 g/mL, and 0.25 g/mL, with methanol as the dilution medium), mobile phase (methanol, ethanol, acetonitrile), flow rate (20 mL/min, 25 mL/min, 30 mL/min, and 35 mL/min), and the mobile-phase composition (92:8, 90:10, 88:12, 86:14, *v*:*v*) were varied for optimization. The performance of the RP-MPLC process was evaluated using the following indicators: the retention time (*t_R_*), resolution from impurities ($R_{S}$), relative purity (*P*), and recovery rate of EPA/DHA ($R_{e}$).

The resolution, a measure of the degree of separation between two adjacent peaks in a chromatogram, reflects an important comprehensive indicator of column efficiency and selectivity. It was calculated according to Equation (2):

(2) $$R_{S}=\frac{2\left|t_{R_{\alpha }}-t_{R_{\beta }}\right|}{W_{1}+W_{2}}\;\times 100\%$$

where Rs (%) represents the resolution between omega-3 fatty acid ethyl esters and impurities, $t_{R_{\alpha }}$(min) represents the retention time of the sample, $t_{R_{\beta }}\;$(min) represents the retention time of impurities, $W_{1}\;$(min) is the peak width of omega-3 fatty acids, and $W_{2}$ (min) is the peak width of impurities.

The recovery rate is the ratio of the content of the target component to its theoretical content in the raw material, and it is an important indicator for measuring separation efficiency. The recovery calculation for the omega-3 fatty acid ethyl ester formula is as follows:

(3) $$R_{e}=\frac{m_{2}\times P_{2}}{m_{1}\times P_{1}}\times 100\%$$

where *R_e_* (%) represents the recovery rate of omega-3 fatty acid ethyl esters as a percentage; *m*_1_ (g) is the mass of the fish oil ethyl esters raw sample, in grams; *m*_2_ (g) is the mass of the purified fish oil ethyl esters, in grams; *P*_1_ (%) is the purity of EPA + DHA in the fish oil ethyl esters raw sample; and *P*_2_ (%) is the purity of EPA + DHA in the purified fish oil ethyl esters.

### 3.5. Comparison of RP-MPLC and RP-HPLC

By using AQ-C18 packing (20–40 μm, 100 Å pore size, 320–340 m^2^/g surface area) as the stationary phase, the effects of RP-MPLC and RP-HPLC chromatography methods on the purification of omega-3 fatty acid ethyl esters were explored based on indicators such as the purity, recovery rate, retention time, operation duration, and solvent consumption.

RP-MPLC conditions: The Pure Chromatography Instrument C-815 Flash system equipped with an AQ-C18 RP-MPLC column (26.2 mm × 152.3 mm) was employed. Sample concentration: pure fish oil ethyl esters; mobile phase: methanol–water (90:10, *v*:*v*); isocratic elution; flow rate: 30 mL/min; UV (wavelength of 210 nm) detectors.

RP-HPLC conditions: The SMB-2–50 Liquid Chromatography System (Hanbon Sci & Tech, Jiangsu, China) fitted with an AQ-C18 RP-HPLC column (10 mm × 150 mm) was utilized. Sample concentration: pure fish oil ethyl esters; mobile phase: methanol–water (90:10, *v*:*v*); isocratic elution; flow rate: 3 mL/min; column temperature: 30 °C; UV detection wavelength: 210 nm.

### 3.6. Statistical Analysis

Samples were analyzed in triplicates, and the data are presented as mean ± standard deviation. One-way analysis of variance (ANOVA) with LSD and Turkey methods and a two-sample *t*-test were carried out using SPSS Statistics 25 (SPSS Inc., Chicago, IL, USA); a difference was considered significant at *p* < 0.05. Graphical analysis was carried out using Origin 2022b software (Origin Lab Corp., Northampton, MA, USA).

## 4. Conclusions

In this study, omega-3 fatty acid ethyl esters with a total content of EPA and DHA of 85.27% and the considerable recovery rate of 74.30% were obtained using AQ-C18 RP-MPLC, the optimal conditions of which were as follows: AQ-C18 packings (20–40 μm, 100 Å pore size, 320–340 m^2^/g surface area), a fish oil sample injection that constituted 1.25% of the column volume; methanol–water (90:10, *v*:*v*) as the mobile phase, isocratic elution at a 30 mL/min flow rate; and an operating pressure of 1–4 bar. To our knowledge, this is the first study to highlight the greater suitability of AQ-C18 packing for purifying omega-3 fatty acids, as it enhanced the retention time via the polar end-capping of the silanol groups, resulting in better purification effects. A comparison between RP-MPLC and RP-HPLC revealed that, although the purity of EPA and DHA ethyl esters produced using both methods met national Pharmacopoeia standards, RP-MPLC allowed for greater sample loading, higher flow rates, and lower system pressure, thereby shortening the purification time, increasing the production efficiency, and reducing production costs. The preliminary RP-MPLC study has demonstrated great potential for industrial production, thereby laying the foundation for pilot-scale experiments. In the future, it will be necessary to conduct pilot-scale experiments to explore the RP-MPLC purification of high-purity omega-3 fatty acids and perform a techno-economic assessment of large-scale production costs.

## Figures and Tables

**Table 1:** Fatty acid composition of the fish oil ethyl esters.

Abbreviation	Compound Name	Content (%)
C14:0	Myristic acid	0.23 ± 0.06
C15:0	Pentadecanoic acid	0.04 ± 0.02
C16:0	Palmitic acid	1.52 ± 0.04
C18:0	Stearic acid	2.18 ± 0.08
C19:0	Nonadecanoic acid	0.10 ± 0.04
C20:0	Arachidic acid	0.37 ± 0.02
C16:1n7	Palmitoleic acid	0.64 ± 0.06
C18:1n9	Octadecenoic acid	4.64 ± 0.05
C20:1n9	11-Eicosenoic acid	1.17 ± 0.03
C22:1n9	Erucic acid	1.19 ± 0.07
C24:1n9	Nervonic acid	0.21 ± 0.03
C18:3n3	α-Linolenic acid	0.54 ± 0.03
C20:3n3	11,14,17-Eicosatrienoic acid	2.43 ± 0.04
EPA C20:5n3	5,8,11,14,17-Eicosapentaenoic acid	40.83 ± 0.12
C21:5n3	Heneicosapentaenoic acid	2.58 ± 0.06
DPA C22:5n3	Docosapentaenoic acid	5.17 ± 0.07
DHA C22:6n3	4,7,10,13,16,19-Docosahexaenoic acid	27.08 ± 0.07
C18:2n6	Linolelaidic acid	0.29 ± 0.03
C18:3n6	Octadecatrienoic acid	0.80 ± 0.04
C18:4n6	Octadecatetraenoic acid	2.79 ± 0.06
C20:3n6	8,11,14-Eicosatrienoic acid	0.52 ± 0.04
C20:4n6	Arachidonic acid	3.46 ± 0.04
C22:4n6	7,10,13,16-Docosatetraenoic acid	0.19 ± 0.02
C22:5n6	4,7,10,13,16-Docosapentaenoate	1.06 ± 0.02
∑SFA	Saturated fatty acids	4.45 ± 0.1
∑MUFA	Mono-unsaturated fatty acids	7.84 ± 0.06
∑PUFA	Poly-unsaturated fatty acids	87.71 ± 0.06
∑ω-3 PUFA	ω-3 Poly-unsaturated fatty acids	78.59 ± 0.23
∑ω-6 PUFA	ω-6 Poly-unsaturated fatty acids	9.12 ± 0.19
EPA + DHA		67.91 ± 0.18

**Table 2:** Effects of AQ-C18 and C18 on the esters of EPA and DHA purified via RP-MPLC.

Packing Materials	*t_R2_* (min)	*t_R3_* (min)	*R_S1_*	*R_S2_*
AQ-C18	17.09 ± 0.08 ^b^	21.53 ± 0.07 ^b^	1.43 ± 0.02 ^a^	1.13 ± 0.03 ^a^
C18	31.08 ± 0.14 ^a^	37.90 ± 0.1 ^a^	1.27 ± 0.03 ^b^	1.02 ± 0.03 ^b^

Note: *t_R2_* represents the retention time of EPA; *t_R3_* represents the retention time of DHA; *R_S1_* represents the resolution of EPA and the preceding impurity (fraction A); *R_S2_* represents the resolution of DHA and the following impurity (fraction D). Different letters within the group indicate significant differences (*p* < 0.05). The same notes apply to the following tables.

**Table 3:** Effects of different mobile phases on the esters of EPA and DHA purified via RP-MPLC.

Mobile Phases	*t_R2_* (min)	*t_R3_* (min)	*R_S1_*	*R_S2_*
Ethanol	6.29 ± 0.08 ^c^	7.14 ± 0.04 ^c^	0	0
Acetonitrile	13.95 ± 0.1 ^b^	15.81 ± 0.08 ^b^	0	1.32 ± 0.02 ^a^
Methanol	17.08 ± 0.06 ^a^	21.54 ± 0.08 ^a^	1.42 ± 0.02 ^a^	1.27 ± 0.03 ^b^

Note: Different letters within the group indicate significant differences (*p* < 0.05).

**Table 4:** Effects of different injection volumes on the esters of EPA and DHA purified via RP-MPLC.

Sample Volumes (mL)	Purity of EPA-EE/DHA-EE (%)	Recovery of EPA-EE/DHA-EE (%)	*t_R2_* (min)	*t_R3_* (min)	*R_S1_*	*R_S2_*
0.4	87.57 ± 0.30 ^a^	58.44 ± 0.13 ^c^	17.10 ± 0.04 ^d^	21.47 ± 0.04 ^d^	1.43 ± 0.02 ^a^	1.07 ± 0.02 ^a^
0.5	86.75 ± 0.08 ^b^	65.43 ± 0.21 ^b^	17.25 ± 0.05 ^c^	21.80 ± 0.03 ^c^	1.32 ± 0.03 ^b^	1.02 ± 0.01 ^b^
0.6	86.67 ± 0.24 ^b^	83.57 ± 0.22 ^a^	17.40 ± 0.05 ^b^	22.07 ± 0.07 ^b^	1.27 ± 0.02 ^b^	1.02 ± 0.02 ^b^
0.7	83.15 ± 0.30 ^c^	63.59 ± 0.36 ^bc^	17.51 ± 0.04 ^a^	22.30 ± 0.06 ^a^	1.06 ± 0.02 ^c^	0.96 ± 0.02 ^c^

Note: Different letters within the group indicate significant differences (*p* < 0.05).

**Table 5:** Effects of different concentrations on the esters of EPA and DHA purified via RP-MPLC.

Sample Concentrations (g/mL)	Purity of EPA-EE/DHA-EE (%)	Recovery Rate of EPA-EE/DHA-EE (%)	*t_R2_* (min)	*t_R3_* (min)	*R_S1_*	*R_S2_*
0.25	87.19 ± 0.19 ^a^	50.47 ± 0.08 ^d^	15.86 ± 0.03 ^c^	18.07 ± 0.06 ^c^	1.38 ± 0.03 ^a^	1.31 ± 0.04 ^a^
0.5	86.63 ± 0.28 ^a^	58.65 ± 0.07 ^c^	17.51 ± 0.04 ^b^	20.69 ± 0.06 ^b^	1.35 ± 0.02 ^b^	1.27 ± 0.03 ^a^
1	86.11 ± 0.11 ^b^	62.21 ± 0.08 ^b^	17.61 ± 0.03 ^b^	21.47 ± 0.04 ^a^	1.29 ± 0.02 ^c^	1.13 ± 0.02 ^b^
Pure	85.75 ± 0.15 ^c^	74.62 ± 0.05 ^a^	17.72 ± 0.02 ^a^	21.92 ± 0.03 ^a^	1.23 ± 0.04 ^c^	1.10 ± 0.03 ^b^

Note: Different letters within the group indicate significant differences (*p* < 0.05).

**Table 6:** Effects of different flow rates on the esters EPA and DHA purified via RP-MPLC.

Flow Rate (mL/min)	Purity of EPA-EE/DHA-EE (%)	Recovery Rate of EPA-EE/DHA-EE (%)	*t_R2_* (min)	*t_R3_* (min)	*R_S1_*	*R_S2_*
20.00	86.17 ± 0.15 ^a^	82.86 ± 0.18 ^a^	17.33 ± 0.06 ^a^	20.60 ± 0.07 ^a^	1.27 ± 0.03 ^a^	1.28 ± 0.04 ^a^
25.00	86.01 ± 0.14 ^a^	76.35 ± 0.01 ^b^	14.08 ± 0.08 ^b^	17.82 ± 0.08 ^b^	1.26 ± 0.03 ^a^	1.06 ± 0.02 ^b^
30.00	85.27 ± 0.15 ^b^	73.82 ± 0.16 ^b^	11.87 ± 0.05 ^c^	15.07 ± 0.06 ^c^	1.23 ± 0.01 ^a^	1.01 ± 0.02 ^b^
35.00	84.16 ± 0.83 ^c^	58.94 ± 0.14 ^c^	9.62 ± 0.09 ^d^	12.09 ± 0.08 ^d^	1.14 ± 0.02 ^b^	0.87 ± 0.03 ^c^

Note: Different letters within the group indicate significant differences (*p* < 0.05).

**Table 7:** Effects of different mobile-phase compositions on the esters of EPA and DHA purified via RP-MPLC.

Methanol–Water (*v*:*v*)	Purity of EPA-EE/DHA-EE (%)	Recovery Rate of EPA-EE/DHA-EE (%)	*t_R2_* (min)	*t_R3_* (min)	*R_S1_*	*R_S2_*
86:14	87.17 ± 0.15 ^a^	54.51 ± 0.16 ^c^	22.81 ± 0.05 ^a^	30.48 ± 0.08 ^a^	1.64 ± 0.04 ^a^	1.41 ± 0.03 ^a^
88:12	86.32 ± 0.10 ^b^	65.24 ± 0.12 ^b^	18.37 ± 0.07 ^b^	24.26 ± 0.06 ^b^	1.50 ± 0.02 ^b^	1.26 ± 0.03 ^b^
90:10	85.27 ± 0.15 ^c^	74.30 ± 0.11 ^a^	11.87 ± 0.05 ^c^	15.07 ± 0.04 ^c^	1.22 ± 0.04 ^c^	1.02 ± 0.02 ^c^
92:8	83.39 ± 0.14 ^d^	53.28 ± 0.01 ^c^	9.67 ± 0.1 ^d^	12.02 ± 0.07 ^d^	1.05 ± 0.03 ^d^	0.84 ± 0.02 ^d^

Note: Different letters within the group indicate significant differences (*p* < 0.05).

**Table 8:** Fatty acid composition of purified omega-3 fatty acid ethyl esters.

Number	Abbreviation	Compound Name	Content (%)
1	C16:0	Palmitic acid	0.19 ± 0.03
2	C18:0	Stearic acid	1.70 ± 0.07
3	C20:0	Arachidic acid	0.51 ± 0.04
4	C16:1n7	Palmitoleic acid	0.53 ± 0.04
5	C18:1n9	Octadecenoic acid	1.40 ± 0.07
6	C20:1n9	Eicosenoic acid	0.61 ± 0.04
7	C22:1n9	Erucic acid	0.57 ± 0.05
8	C18:3n3	α-Linolenic acid	0.58 ± 0.05
9	C20:3n3	11,14,17-Eicosatrienoic acid	2.20 ± 0.03
10	C20:5n3	5,8,11,14,17-Eicosapentaenoic acid	57.13 ± 0.08
11	C21:5n3	Heneicosapentaenoic acid	2.29 ± 0.04
12	C22:6n3	4,7,10,13,16,19-Docosahexaenoic acid	28.14 ± 0.08
13	C18:2n6	Linoleic acid	0.43 ± 0.19
14	C18:3n6	Octadecatrienoic acid	0.46 ± 0.04
15	C18:4n6	Octadecatetraenoic acid	0.18 ± 0.06
16	C20:4n6	Arachidonic acid	3.08 ± 0.07
17	∑SFA	Saturated fatty acids	2.40 ± 0.08
18	∑MUFA	Mono-unsaturated fatty acids	3.14 ± 0.07
19	∑PUFA	Poly-unsaturated fatty acids	94.47 ± 0.15
20	∑ω-3 PUFA	ω-3 Poly-unsaturated fatty acids	90.34 ± 0.17
21	∑ω-6 PUFA	ω-6 Poly-unsaturated fatty acids	4.13 ± 0.15
22	EPA + DHA		85.27 ± 0.15

**Table 9:** Comparison of purification effects of RP-MPLC and RP-HPLC methods on omega-3 fatty acid ethyl esters.

Methods	Purity of EPA-EE/DHA-EE (%)	Recovery Rate of EPA-EE/DHA-EE (%)	*t_R2_* (min)	*t_R3_* (min)	Duration (min)	Solvent Consumption (mL)
RP-MPLC	85.27 ± 0.15 ^b^	74.30 ± 1.14 ^b^	11.87 ± 0.07 ^b^	15.08 ± 0.06 ^b^	22.17 ± 0.76 ^b^	665 ± 20.91 ^a^
RP-HPLC	86.11 ± 0.96 ^a^	78.56 ± 0.86 ^a^	28.51 ± 0.06 ^a^	41.27 ± 0.07 ^a^	75 ± 1.05 ^a^	225.50 ± 3.18 ^b^

Note: Different letters within the group indicate significant differences (*p* < 0.05).

## Data Availability

The data presented in this study are available on request from the corresponding author.
